# Injection of embryonic stem cell derived macrophages ameliorates fibrosis in a murine model of liver injury

**DOI:** 10.1038/s41536-017-0017-0

**Published:** 2017-05-23

**Authors:** Sharmin S. Haideri, Alison C. McKinnon, A. Helen Taylor, Phoebe Kirkwood, Philip J. Starkey Lewis, Eoghan O’Duibhir, Bertrand Vernay, Stuart Forbes, Lesley M. Forrester

**Affiliations:** Centre for Regenerative Medicine, University of Edinburgh, 5 Little France Drive,, Edinburgh, EH16 4UU UK

## Abstract

Chronic liver injury can be caused by viral hepatitis, alcohol, obesity, and metabolic disorders resulting in fibrosis, hepatic scarring, and cirrhosis. Novel therapies are urgently required and previous work has demonstrated that treatment with bone marrow derived macrophages can improve liver regeneration and reduce fibrosis in a murine model of hepatic injury and fibrosis. Here, we describe a protocol whereby pure populations of therapeutic macrophages can be produced in vitro from murine embryonic stem cells on a large scale. Embryonic stem cell derived macrophages display comparable morphology and cell surface markers to bone marrow derived macrophages but our novel imaging technique revealed that their phagocytic index was significantly lower. Differences were also observed in their response to classical induction protocols with embryonic stem cell derived macrophages having a reduced response to lipopolysaccharide and interferon gamma and an enhanced response to IL4 compared to bone marrow derived macrophages. When their therapeutic potential was assessed in a murine, carbon tetrachloride-induced injury and fibrosis model, embryonic stem cell derived macrophages significantly reduced the amount of hepatic fibrosis to 50% of controls, down-regulated the number of fibrogenic myofibroblasts and activated liver progenitor cells. To our knowledge, this is the first study that demonstrates a therapeutic effect of macrophages derived in vitro from pluripotent stem cells in a model of liver injury. We also found that embryonic stem cell derived macrophages repopulated the Kupffer cell compartment of clodronate-treated mice more efficiently than bone marrow derived macrophages, and expressed comparatively lower levels of *Myb* and *Ccr2*, indicating that their phenotype is more comparable to tissue-resident rather than monocyte-derived macrophages.

## Stem cell-derived macrophages could treat liver fibrosis

Macrophages, a type of white blood cell, when derived from embryonic stem cells in the laboratory reduce fibrosis in chronic liver disease. Lesley Forrester and colleagues from the University of Edinburgh found murine embryonic stem-cell-derived macrophages (ESDM) were morphologically similar to bone marrow-derived macrophages (BMDM), previously found to reduce fibrosis and improve liver function in mice with induced liver injury. Using a novel technique, the team found ESDM engulfed fewer particles at a slower rate than BMDM, indicating ESDM were less inflammatory. A higher dose of ESDM was required to have the same effect of BMDM to help liver fibrosis regression. However, they were more efficient in repopulating mouse livers depleted of liver-specific macrophages and also significantly improved liver function, indicating ESDM were similar to resident macrophages in the liver and had therapeutic potential.

## Introduction

The regeneration of tissue after injury or disease involves the complex interplay between stem cells that are capable of directly regenerating that organ and a variety of support cells that constitute their niche. Therapies to promote regeneration involve both direct cell replacement and the modification of the environment to promote endogenous repair.^1^

Stem cells in the healthy liver have an enormous capacity for regeneration but chronic injury induced by viral infection, alcohol abuse, exposure to toxins and metabolic disorders leads to fibrotic scaring, cirrhosis and the ultimate failure of that regenerative process.^2^ The only treatment available is organ transplantation but with increasing demand and a limited number of donors, there is an urgent requirement for alternative therapies. Strategies involving the transplantation of mature hepatocytes has not been particularly successful^3^ indicating that it is also crucial to remodel and repair the damaged niche. Cellular therapies that aim to have an anti-fibrotic or pro-regenerative effect have met with some success. Bone marrow cell transplantation can influence the progression and recovery of liver fibrosis with more recent studies indicating that the monocyte/macrophage lineage is the key cell mediator of this effect.^4, 5^ The delivery of bone marrow-derived macrophages (BMDMs) during carbon tetrachloride (CCl_4_)-induced liver injury significantly reduced the amount of fibrosis and improved liver function.^5^ Also, human macrophages delivered to an immunocompromised mouse during CCl_4_-induced liver injury improved liver function and reduced fibrosis.^6^ Macrophages aid in fibrosis resolution via phagocytosis of debris and also by secreting matrix metalloproteases (MMPs) that break down extracellular matrix components of scar tissue.^7^ Depletion of restorative macrophages during the resolution of fibrosis in mice was shown to cause a failure in scar remodeling.^8^

Macrophages are thought to stimulate ductular cells to proliferate through the secretion of tumor necrosis factor-related weak inducer of apoptosis (TWEAK).^9, 10^ These ductular cells may be associated with fibrosis^11^ but may contain regenerative hepatic progenitor cells.^12^ Therefore macrophages may have therapeutic potential both by reducing fibrosis and by stimulating liver regeneration.

Further studies on the molecular mechanisms involved in macrophage tissue repair requires a strategy, whereby a consistent supply of cells can be genetically modified to deplete key molecular components. Furthermore, although autologous monocytes are a potential source of regenerative macrophages, a source that has the potential for an “off the shelf” cell therapy would be essential for the treatment of acute disease. Combining the proliferative and differentiation capacity of pluripotent stem cells (PSCs) and the arsenal of genetic engineering technologies available for their genetic manipulation provides the necessary tools to progress. Previous studies have demonstrated that it is possible to produce macrophages from mouse and human PSCs^13, 14^ with an in depth RNAseq analysis indicating a broadly similar transcriptional profile between human monocyte-derived and iPSC-derived macrophages.^15^

We demonstrate that pure populations of macrophages can be produced from mouse embryonic stem cells (ESCs) in vitro at scale and that these cells have the capacity for repair in vivo in a murine model of liver fibrosis. ESC-derived macrophages can be distinguished from their bone marrow derived counterparts in phagocytic activity and in the expression of key functional markers supporting the recent study suggesting that PSC-derived macrophages are developmentally related to tissue resident macrophages including Kupffer cells and Langerhans cells.^16^ We show that ESC-derived macrophages are able to repopulate the Kupffer cell compartment of macrophage-depleted livers more efficiently than BMDMs and thus provide an effective strategy to study the role of tissue resident macrophages in tissue repair.

## Results

### A comparison of embryonic stem cell derived-macrophages and bone marrow derived macrophages

**Fig. 1:**
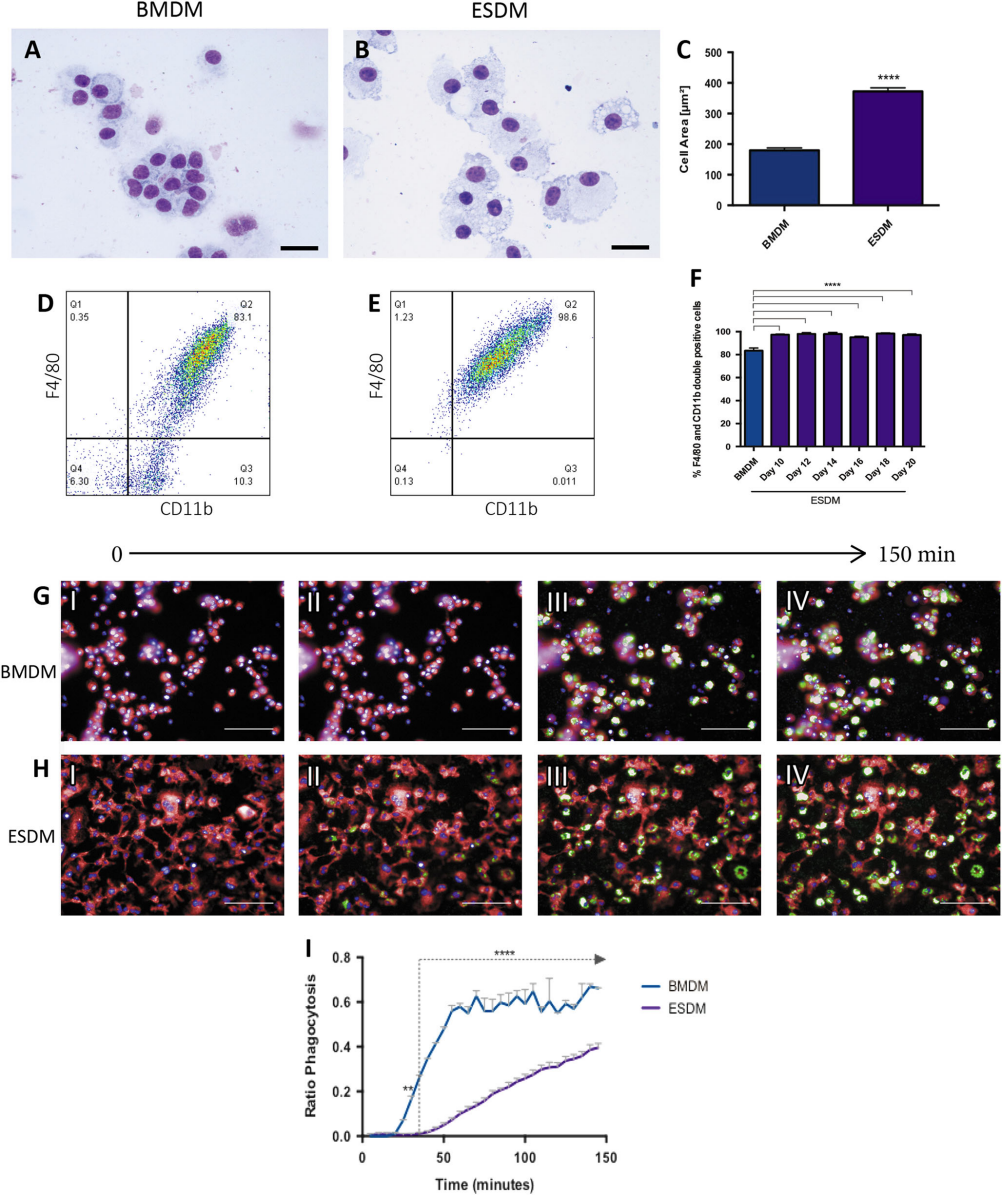
Comparison of ESDMs and BMDMs. Stained cytospins of BMDMs (**a**) and ESDMs (**b**) with image analyses demonstrating that ESDMs are larger (**c**) (*Scale bars* 30 μM). Flow cytometry analyses of BMDMs (**d)** and ESDM (**e**) demonstrating a pure population in ESDMs. Images from live phagocytosis assay of BMDMs (**g**) and ESDMs (**h**) at 0 (I), 50 (II, 100 (III) and 150 (IV) minutes after the addition of Phrodo beads and quantification of rate of phagocytosis using Harmony image analysis software (**i**) (*Scale bars* 50 μM). See Supplementary Fig. S1 for details of the assay. [*p < 0.05, **p < 0.01, ***p < 0.001, ****p < 0.0001; *n* = 3, **c** Unpaired t-test, **f** One-way ANOVA with Kruskal-Wallis test, **i** Two-way ANOVA with Sidak’s multiple comparison test]

We produced macrophages from ESC using our published method^13^ and directly compared their phenotype and function with classic BMDMs, the gold-standard source of primary macrophages. Briefly, ESC were grown in the presence of CSF-1 and IL-3 to form embryoid bodies (EB). EBs adhere to tissue culture plastic and release non-adherent macrophage progenitor cells into the medium. These cells are harvested and plated onto non-treated Petri dishes in the presence of CSF-1 alone. They give rise to macrophages that adhere to the plastic and form a monolayer. Embryonic stem cell derived macrophages (ESDMs) and BMDMs had a comparable morphology but ESDMs appeared slightly larger in size than BMDMs (Fig. 1a–c). Flow cytometry analyses revealed that over 90% of ESDMs were double positive for the macrophage markers, F4/80 and CD11b, and that cells derived from different time points during the differentiation process (day 10–20) demonstrated comparable levels of cell surface marker expression (Fig. 1e, f). In comparison, the population of BMDMs had a slightly lower percentage of double positive F4/80 and CD11b cells indicating BMDMs generated in this protocol were more heterogeneous than ESDMs (Fig. 1d and f).

To compare the phagocytic activity of ESDMs and BMDMs, we developed a novel live imaging assay using the operetta high-content imaging system (Perkin Elmer) which is fully automated and thus eliminates operator bias out of imagining and analysis. In this assay pH-sensitive pHrodo^TM^ Bioparticles were used that only fluoresce within the acidic intracellular environment (Thermo Fisher Scientific). This assay allowed us to quantify amount as well as rate of uptake of bioparticles in real time. Cells were stained with a deep red plasma membrane stain and NucBlue Live ReadyProbes reagent, incubated with pHrodo^TM^ bioparticles and imaged every 5 min for 2.5 h using the operetta high-content system (Fig. 1g, h) (see videos in Supplementary Figs S5–7 and Supplementary Fig. S1). An increase in the number of fluorescing cells was observed over time in both cell types but the phagocytosis rate of ESDMs was significantly lower than BMDMs (Fig. 1i). Our previous work using flow cytometry indicated that there was no significant difference in phagocytic activity between BMDMs and ESDMs whereas this sensitive, real time strategy has uncovered subtle differences.

**Fig. 2:**
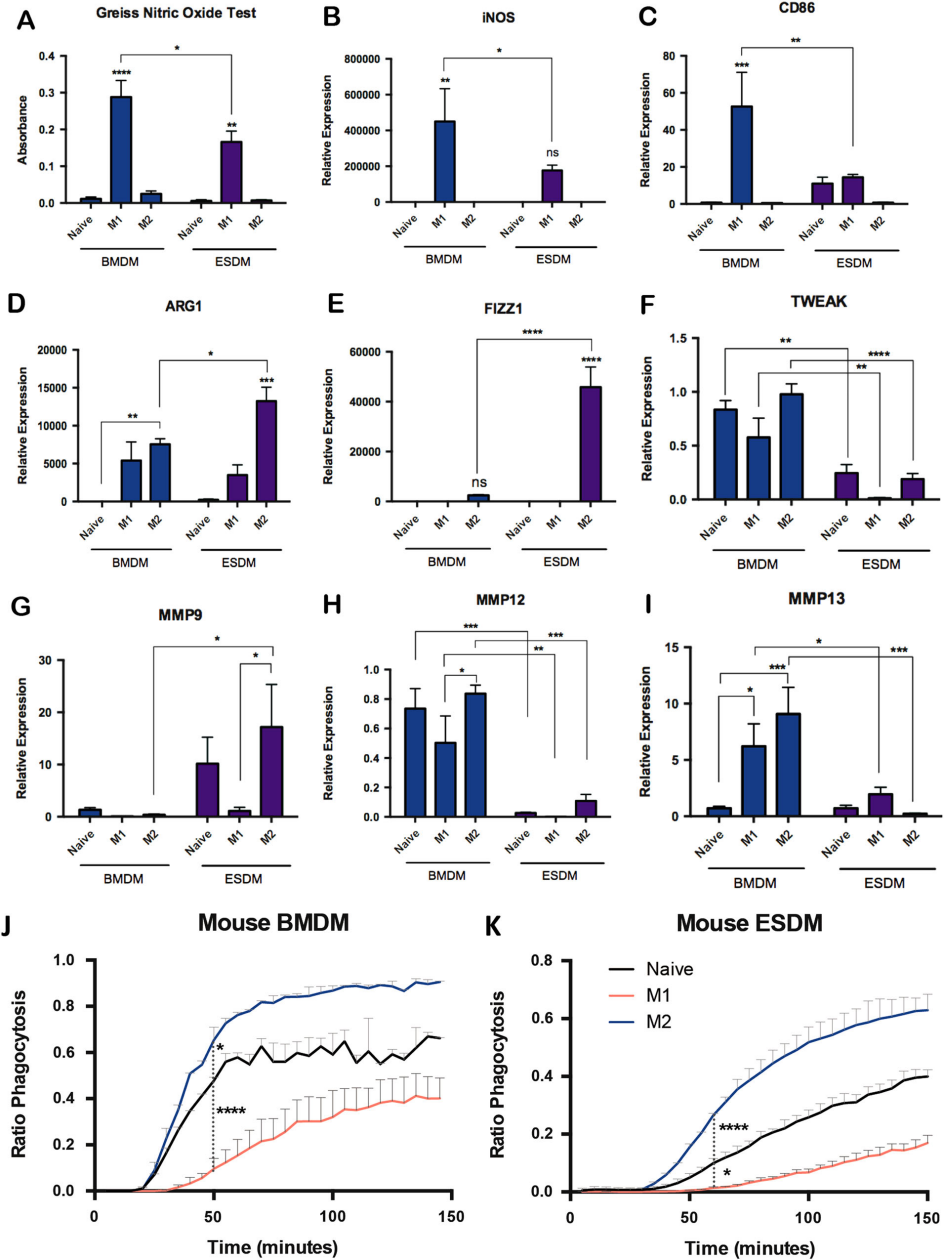
M1 and M2 stimulation of ESDMs and BMDMs. Griess nitric oxide assay of naïve, M1 and M2-stimulated BMDMs and ESDMs (**a**), and expression of key markers by qRT-PCR analyses, *iNos* (**b**) and *Cd86* (**c**) as markers of M1, *Arg1* (**d**) and *Fizz1* (**e**) as markers of M2 phenotypes, and *Tweak* (**f**), *Mmp9* (**g**), *Mmp12* (**h**), and *Mmp13* (**i**) as markers of tissue remodeling. Quantification of phagocytosis of M1-and M2-stimulated BMDMs (**j**) and ESDMs (**k**). [*p < 0.05, **p < 0.01, ***p < 0.001, ****p < 0.0001; *n* = 3 per group, Two-way ANOVA with Tukey’s multiple comparison test]

ESDMs and BMDMs were primed in vitro to adopt a M1-like or M2-like phenotype by treating them with lipopolysaccharide (LPS) and interferon gamma (IFNγ) or IL-4, respectively. As expected, there was a significant increase in nitric oxide (NO) production upon LPS/IFNγ-stimulation from both BMDM and ESDMs but the activation level of ESDMs was not as high as BMDMs (Fig. 2a). This was confirmed by the lower level of expression of M1-related genes, *iNos* and *Cd86* (Fig. 2b, c) in ESDMs. In contrast, the response of ESDMs to IL4 was significantly higher than BMDMs as assessed by the increased expression of the M2-related genes, *Arg1* and *Fizz1* (Fig. 2d, e). Markers of tissue regeneration such as *Tweak*, *Mmp12*, and *Mmp13* were lower in ESDMs compared to BMDM whereas *Mmp9* was expressed at a higher level (Fig. 2f–i).

We used our novel imaging strategy to compare the phagocytic activity of M1-polarized and M2-polarized ESDM and BMDM. LPS-IFNγ stimulation reduced and IL4 increased the rate of phagocytosis in both cell types but, ESDMs had a lower rate of phagocytosis in their stimulated cultures compared to the equivalent cultures of BMDMs (Fig. 2j, k).

Taken together these data demonstrate that the phenotype of ESDMs can be modified in a comparable manner to BMDMs but subtle differences in the extent of their response to polarization indicates that they have a less inflammatory phenotype.

**Fig. 3:**
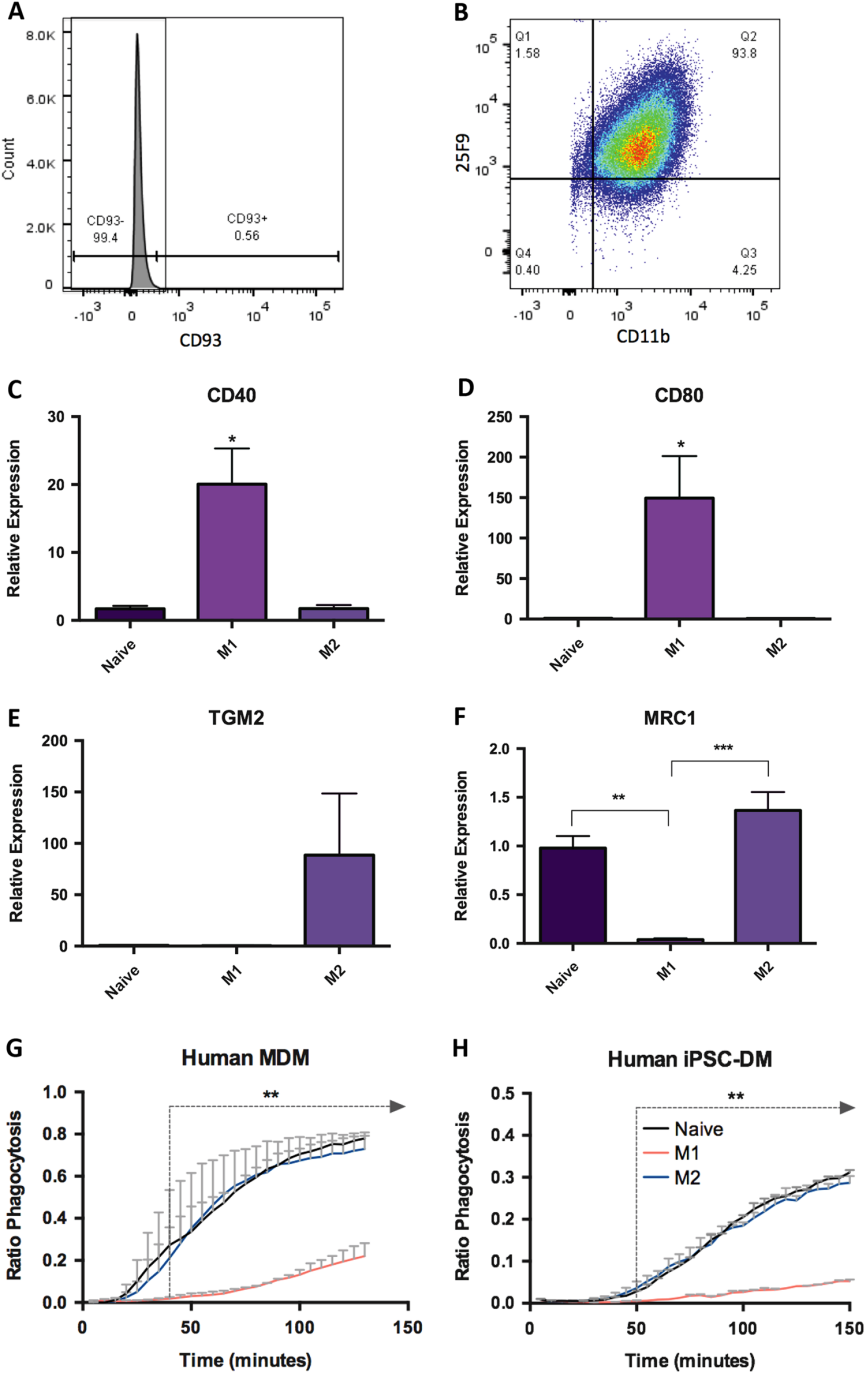
Production of macrophages from human PSCs. Macrophage derived from hPSC express both CD11b and 25F9 (**b**) but not CD93 (**a**) and key M1 (**c**, **d**) and M2-associated genes (**e**, **f**) are upregulated upon stimulation. Assessed using a live imaging phagocytosis assay, naïve and M2-activated macrophages were more phagocytic than M1-activated macrophages and this was observed for both MDMs (**g**) and iPSC-DMs (**h**). [*p < 0.05, **p < 0.01, ***p < 0.001; *n* = 3, **c**–**f** One-way ANOVA, **g**, **h** Two-way ANOVA with Tukey’s multiple comparison test]

To extend our work to human studies, we optimized a protocol to generate macrophages from human induced pluripotent stem cells (iPSC) based on work published by Cowley et al.^17^ The human iPSC-derived macrophages (iPSC-DM) that were generated expressed both the mature macrophage-specific cell surface marker 25F9 and CD11b (Fig. 3b) and did not express the monocyte marker CD93 (Fig. 3a). Human iPSC-DM responded to stimuli as expected: M1-related genes *CD40* and *CD80* were upregulated when activated by LPS-IFNγ, whereas M2-related genes *TGM2* and *MRC1* gene expression were elevated in response to IL4 (Fig. 3c–f).

We compared naïve and polarized human iPSC-DM with human monocyte-derived macrophages (MDM) in our live imaging phagocytosis assay. Human iPSC-DM followed the same trend as human MDM, where “naïve” and IL4-treated macrophages were found to be more phagocytic then LPS-IFNγ-treated macrophages (Fig. 3g and h). However, reminiscent of our observations in mouse ESDM, human iPSC-DM phagocytosed less than human MDM in their “naïve” as well as polarized states indicating that they have a less inflammatory phenotype.

### ESDMs as an effective cell therapy in a murine model of liver fibrosis

**Fig. 4:**
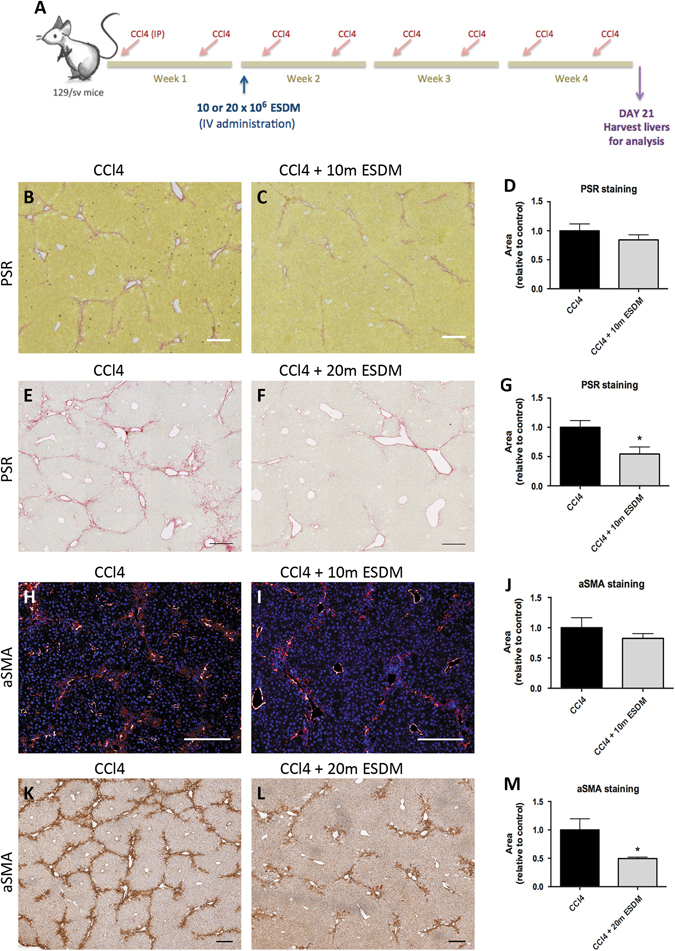
The higher dose of ESDMs has an anti-fibrotic effect. Schematic **a** of injury and macrophage injection of 10 or 20 million (m) macrophages. PSR staining (**b**–**g)** and aSMA staining (**h**–**m)** of liver sections from CCl_4_-treated livers that were injected with either 10 million (10 m) or 20 million (20 m) ESDMs. *Scale bars* 200 μM. [*p < 0.05, *n* = 5 per group, Unpaired *t*-test]

We next tested whether ESDMs could have a therapeutic effect in a murine model of liver injury. Mice were treated with CCl_4_ twice weekly for 4 weeks with a proportion being randomly selected to receive either 10 or 20 × 10^6^ ESDM intravenously at the start of the second week. Mice were sacrificed 21 days after ESDM injection and livers were processed for immunohistochemistry (Fig. 4a).

BMDM therapy had previously been shown to ameliorate fibrosis so we assessed whether ESDMs also had an effect on hepatic fibrosis using picro sirius red (PSR) stain for hepatic collagens. When the lower number (10 × 10^6^) of ESDM was used there was no significant effect on the level of fibrosis (Fig. 4b–d) whereas when twice as many ESDMs were injected, a significant reduction in PSR staining was observed (Fig. 4e–g). A decline in the number of myofibroblasts is a key event during fibrosis resolution and we also noted a significantly lower number of αSMA positive myofibroblasts in mice treated with the higher dose of ESDMs (Fig. 4k–m). Healthy, uninjured livers was also stained and analyzed (Supplementary Fig. S2).

**Fig. 5:**
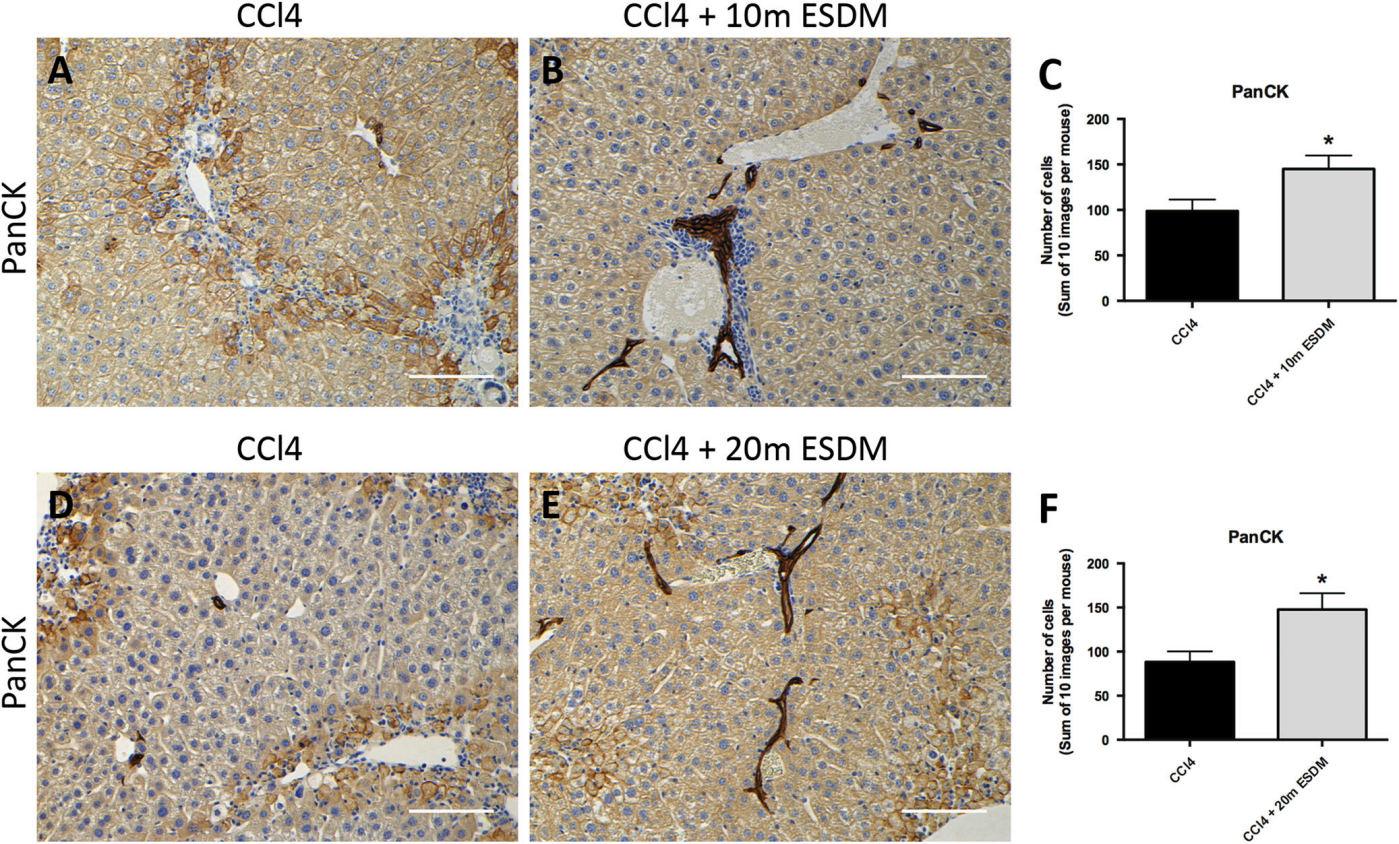
ESDMs can initiate a progenitor response in injured livers. A significantly higher number of PanCK-positive cells were observed in mice injected with ESDMs compared to control injury livers (**a**–**f**). *Scale bars* 100 μM. [*p < 0.05, *n* = 5 per group, Unpaired *t*-test]

We observed a significantly higher number of hepatic ductular cells, marked by PanCK staining, in CCl_4_-injured livers that had been injected with ESDMs compared to control injured livers (Fig. 5a–f). The higher number of PanCK-positive cells was evident in animals receiving both doses of ESDMs replicating an effect previously observed with BMDMs.^9^

In conclusion this study demonstrates that ESDMs can be produced on a large scale and they have the potential to modulate the effect of liver injury by reducing the density of myofibroblasts and resultant collagen deposition. It is important to note that the cell number of ESDM used in these experiments was 10-fold higher than that used for BMDMs in previous studies indicating that ESDMs could be less potent than BMDMs in their reparative activity. Based on a large number of experiments using BMDMs^5, 9^ we are confident about the number of BMDMs that are required to evoke a response but a direct comparison of ESDMs and BMDMs would have to be performed to verify the differences in potency.

### Do ESDMs have a phenotype comparable to tissue resident macrophages?

**Fig. 6:**
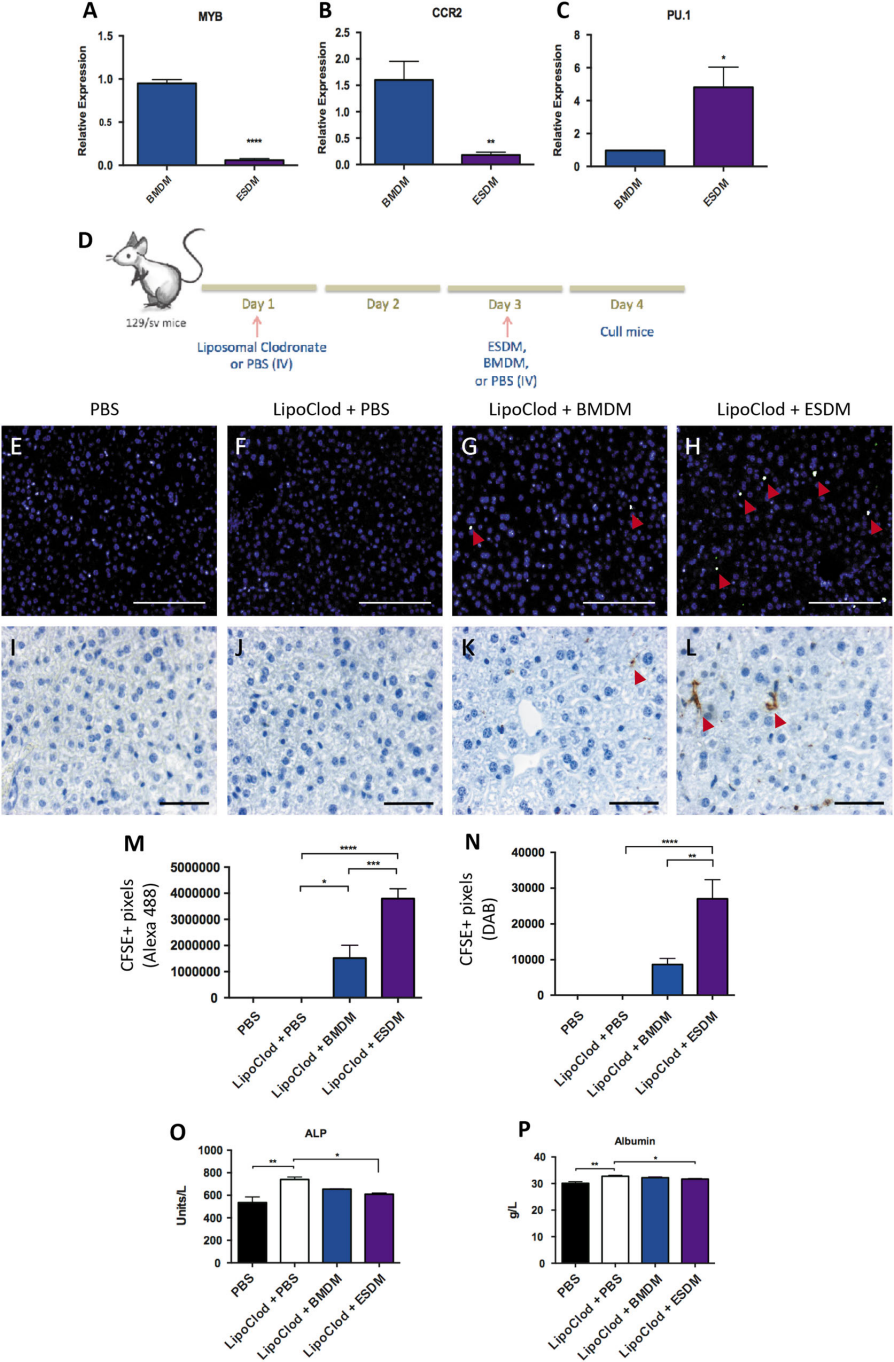
ESDMs can repopulation the liver of liposomal chlodronate-treated mice. Expression of markers differentially expressed in monocyte-derived vs. tissue-resident macrophages, *Myb* (**a**), *Ccr2* (**b**), and *Pu.1* (**c**). Schematic representation (**d**) of liposomal clodronate-induced Kupffer cell depletion and injection of ESDM or BMDM. CFSE immunostaining (**e**–**h** *Scale bars* 100 μM) and CFSE immunohistochemistry (**i**–**l** *Scale bars* 50 μM.) of liver sections from the different treatment groups. Quantification of CFSE+ immunostaining (**m**) and CFSE+ immunohistochemistry (**n**). Arrowheads point at CFSE+ cells detected. Blood serum analysis of **o** Alkaline phosphatase (ALP), and **p** Albumin levels. [*p < 0.05, **p < 0.01, ***p < 0.001, ****p < 0.0001; *n* = 5 per group, One-way ANOVA]

It is widely accepted that haematopoietic cells derived from ESCs in vitro are more related to the blood cells associated with the primitive wave of haematopoiesis in the yolk sac during development.^18^ Tissue resident macrophages are known to be derived from the primitive wave of haematopoiesis, well before the appearance of haematopoietic stem cells (HSC) in the embryo and develop in the absence of Myb, an important HSC transcription factor.^19^ We considered that the subtle differences that we observed between murine ESDM and BMDMs could be related to their source rather than their culture conditions as suggested in a recent study of human iPSC-DM.^16^ We confirmed this by demonstrating that *Myb* and *Ccr2* are expressed at a lower level in ESDMs compared to BMDMs, whereas *Pu.1* was higher (Fig. 6a–c). Thus if ESDMs are more like tissue resident macrophages than circulating monocyte-derived cells, we hypothesized that they would be more efficient at repopulating the Kupffer cell compartment of the liver following ablation of endogenous resident macrophages with liposomal clodronate.

We first confirmed that liposomal clodronate treatment did indeed ablate the resident macrophage population and analyzed the tissue at different time-points to determine the optimal time for the addition of exogenous macrophages. No F4/80^+^ macrophages were observed in liver sections 48 h after liposomal clodronate treatment indicating that complete depletion had occurred by this time point. A few F4/80^+^ macrophages were evident 72 h after treatment when endogenous circulating macrophages begin to repopulate the tissue (Supplementary Fig. S3).

ESDM and BMDM were labeled with CFSE cell tracking dye (Molecular probes, Life Technologies) and 5 × 10^6^ cells were injected intravenously 48 h after liposomal clodronate treatment (Fig. 6d). Liver, lung, kidney, and heart tissue was harvested 24 h later and immunostaining using an anti-FITC antibody (which detects all derivatives of fluorescein including CFSE) was used to detect the injected CFSE-stained cells (Fig. 6e–h, Supplementary Fig. S4). CFSE^+^ cells were detected in liver sections derived from liposomal clodronate treated mice that received either BMDM or ESDM. Quantification of images demonstrated that there were significantly more CSFE^+^ cells in the livers of mice that were injected with ESDMs compared to BMDMs (Fig. 6m). This result was confirmed by quantification of a DAB-based detection method to identify CSFE^+^ cells (Fig. 6i–l, n). Some BMDMs and ESDMs were also detected in lung tissue but very few cells engrafted in the kidney and heart of clodronate treated animal. (Supplementary Fig. S4).

These data indicate that ESDMs have a higher capacity to repopulate the Kupffer cell compartment than BMDMs which could suggest that ESDMs have a phenotype more akin to tissue resident macrophages.

Serum analysis was carried out to assess the effects of the treatments carried out on liver function of the mice (Fig. 6o, p). Alkaline phosphatase (ALP) levels were significantly elevated in liposomal clodronate treated mice compared to phosphate buffered saline (PBS) control mice indicating liver damage. ALP levels were significantly lowered in mice that received ESDM, compared to controls indicating that ESDMs had some reparative effect. Serum albumin levels of ESDM recipient mice were also comparable to healthy controls.

## Discussion

We have compared the phenotype and functional properties of ESDM and BMDM. The activation profile of ESDM indicates that they are skewed towards an anti-inflammatory phenotype. Unstimulated ESDM had elevated levels of M2-related genes and they did not mount the same level of response to inflammatory stimuli as BMDM. Our findings are consistent with previous publications that demonstrated that ESDM are less inflammatory and more M2-like in comparison to BMDM.^20^

We developed a live imaging assay to evaluate macrophage phagocytic ability. This allowed us to monitor not only the number of particles engulfed at the end, but also the rate at which macrophages phagocytosed the particles. We found that unstimulated ESDM did not phagocytose as fast or as many bioparticles as BMDM. Furthermore, ESDM and BMDM primed towards the M2-like phenotype phagocytosed more bioparticles than their M1-primed equivalents. Our results corroborate with findings from previous studies showing that ESDM are less phagocytic than BMDM.^20^

Our in vitro characterization lead us to hypothesize that ESDMs would prove to be more beneficial for fibrosis resolution as they were skewed towards an anti-inflammatory phenotype, and did not respond as much as BMDMs to inflammatory stimuli.

### ESDM aids fibrosis regression in a murine model of liver fibrosis

When 20 × 10^6^ ESDM were transplanted, the amount of liver fibrosis was significantly reduced and the number of αSMA + myofibroblasts was reduced to 50% of control after ESDM delivery. No significant changes were observed in fibrosis or myofibroblast numbers when 10 × 10^6^ ESDM were transplanted indicating that this anti-fibrotic effect was dose dependent. Interestingly, like BMDMs, both high and low doses of ESDMs stimulated a ductular response as demonstrated by a significant increase in PanCK + progenitors. These data demonstrate that ESDM therapy can aid in regeneration of damaged liver tissue by inducing the proliferation of PanCK + ductular cells, and at high dose, ameliorate fibrosis. To our knowledge, this is the first study that demonstrates a therapeutic effect of ESDMs in a model of liver injury.

The number of ESDMs required to result in a therapeutic effect was much higher than BMDMs. In a previous study, Thomas et al. showed that 1 × 10^6^ transplanted BMDMs had a therapeutic effect during liver fibrosis.^5^ Whereas in our study 20 × 10^6^ ESDMs were required to exert a comparable response.

We had found earlier that ESDMs were less phagocytic than BMDMs in a live imaging phagocytosis assay. This led us to postulate that ESDMs probably did not phagocytose as much as BMDMs in vitro and therefore less fibrosis resolution was observed. And more ESDMs were required to have an effect comparable to 1 × 10^6^ BMDMs.

### ESDM are more efficient at repopulating a Kupffer cell-depleted liver than BMDM

The above results led us to propose that ESDM may be similar to tissue resident macrophages instead of MDM as recently suggested for human iPSC-derived macrophages.^16^ Studies have shown that CSF-1 promotes a resident macrophage phenotype and regulates tissue-resident macrophage numbers.^21^ In our study, ESC were differentiated in the presence of CSF-1 to produce ESDM and it is possible that this promotes a resident phenotype.

We found that ESDMs expressed lower levels of *Myb* and *Ccr2* and higher expression of *Pu.1* than BMDMs, indicating a tissue-resident macrophage profile. Therefore we assessed the repopulation capacities of ESDM and BMDM in vivo in Kupffer cell depleted livers to test if ESDM would be more efficient than BMDM in this experiment. Significantly higher numbers of CFSE + transplanted cells were detected in ESDM recipients compared to BMDM recipients. These results indicated that ESDM were more efficient than BMDM at reversing the effects of liposomal clodronate treatment in mice by partially repopulating the Kupffer cell compartment. This could either be due to the fact that the phenotype of ESDMs is more comparable to a tissue resident macrophage than BMDMs or that they have an enhanced ability to migrate to the macrophage-depleted tissue.

A study comparing human ESC-derived macrophages (hESC-DM) (generated in the presence of CSF-1 and IL-3), fetal liver-derived macrophages and adult monocyte derived macrophages, showed that hESC-DM and fetal liver macrophages followed a similar differentiation pathway, distinct from that of MDM. hESC-DM and fetal liver macrophages were also found to be M2 polarized.^22^

In conclusion, we were able to produce a homogenous population of functional macrophages from mouse and human PSCs. They exhibited characteristic macrophage morphology and expressed macrophage specific cell surface markers. When compared to mouse BMDM and human MDM respectively, they were found to be less phagocytic and skewed towards an anti-inflammatory phenotype. When mouse ESDM were assessed in a murine CCl4-induced model of liver fibrosis, they helped in fibrosis regression, reduced the number of activated myofibroblasts and induced the proliferation of hepatic ductular cells. However, a higher number of ESDM were required (compared to BMDM) to have that effect. When we compared their abilities in repopulating the resident Kupffer cell compartment in liposomal clodronate treated livers, ESDM were found to be more efficient than BMDM at repopulating Kupffer cell-depleted livers, and also significantly improved their liver function. This indicates that murine ESDM have a phenotype more akin to tissue resident macrophages rather than circulating monocyte derived macrophages which supports recent findings in the human system.^16^ We show for the first time that macrophages derived from ESC are functionally therapeutic for chronic liver disease.

As a first step towards translating the therapeutic effects of macrophages in a clinical setting, we optimized a feeder-free and serum-free protocol to efficiently generate macrophages from human iPSC.

## Materials and methods

### Mouse ESC maintenance and differentiation

The mouse ESC line E14IV was maintained in Glasgow minimum essential medium (Gibco) supplemented with 10% fetal calf serum (FCS) (Lonza), 2 mM Sodium pyruvate (Gibco), 4 mM l-glutamine (Gibco), 1% non-essential amino acids (Gibco) and 0.1 mM β-mercaptoethanol (Sigma) and 100 U/mL of leukemia inhibitory factor (LIF) as previously described.^23^ The same medium (without LIF) supplemented with 15% L929-conditioned media and 1 ng/mL recombinant IL-3 (Stem Cell Technologies) (herein referred to as Differentiation medium) was used for the production of macrophage as described previously (Zhang et al.). Briefly, 6 × 10^5^ ESCs were cultured in suspension in differentiation medium to form EB. On day 8, EBs were plated onto gelatinised tissue-culture dishes then non-adherent cells were harvested from these cultures on alternate days (day 10–20) then plated onto non-treated bacteriological petri dishes in differentiation medium without IL-3. These monocyte-like cells adhered to the plastic petri-dishes forming a monolayer and matured into ESDM.

### Bone marrow derived macrophages

Bone marrow was flushed from murine femurs and tibia, cultured in Ultra-low attachment 25 cm^2^ flasks (Corning) in Dulbecco’s Modified Eagle Medium (DMEM) (Sigma) with 10% FCS, 15% L929-conditioned media and 1% Pen/Strep for 7 days (with a media change on day 4) then BMDM were harvested by collecting all adherent and non-adherent cells.

### Differentiation of human iPSCs to macrophages

The human induced PSCs line, SFCi55 was generated in house and was confirmed to be pluripotent and have a normal karyotype.^24^ They were maintained in StemPro medium prepared by supplementing DMEM/F12 with Glutamax (Invitrogen) with StemPro supplement (Invitrogen), 1.8% BSA (Invitrogen), 0.1 mM β-mercaptoethanol (Invitrogen) and 20 ng/mL human basic fibroblast growth factor (Invitrogen). The method for differentiation of iPSCs to macrophages was adapted from Cowley et al.^17^ On Day 0, spent medium was removed from one confluent well of a 6-well plate, and replaced with 2 mL StemPro (ThermoFisher) supplemented with cytokine Mix 1 (50 ng/mL BMP4, 50 ng/mL VEGF, and 20 ng/mL SCF). Cells were cut using the EZPassage^TM^ tool, and gently dislodged with a Pasteur pipette. They were divided equally into two wells of an ulta-low attachment 6-well plate (Corning), and 2 mL X-VIVO^TM^ 15 media with cytokine Mix 1 was added to each well. Cells were cultured in suspension for 3 days with a cytokine top up on Day 2, to make EBs. On Day 4, EBs were lifted and transferred to gelatin-coated tissue-culture grade 6-well plates in X-VIVO^TM^ 15 media supplemented with cytokine Mix 2 (100 ng/mL M-CSF, 25 ng/mL IL3, 2 mM Glutamax, 1% Penicillin/Streptomycin, 0.055 M β-mercaptoethanol). Approximately 30 EBs were plated in each well. EBs were maintained in this medium for the remaining duration of the protocol, with spent medium being replaced with fresh medium every 3–4 days. After about 2 weeks, the EBs produced macrophage progenitors in the culture supernatant that were harvested and transferred to 10 cm^2^ bacteriological dishes in X-VIVO^TM^ 15 medium supplemented with cytokine Mix 3 (100 ng/mL M-CSF, 2 mM Glutamax, 1% Penicillin/Streptomycin) and allowed to mature for 5-7 days into iPSC-DM. Macrophage progenitors were harvested twice a week for approximately 2 months.

### Cytospin and rapid romanowsky staining

Cytospins of macrophages were prepared by harvesting, counting and re-suspending 1 × 10^5^ macrophages in 200 μL PBS. They were cytocentrifuged at 300×*g* for 3 min in a Thermo Shandon Cytospin 4 and allowed to air dry. The Rapid Romanowsky Stain Pack (TCS Biosciences) was used to fix and stain the cells as per manufacturer’s instructions. The slides were rinsed briefly in water, air-dried and mounted with Mowiol (Sigma).

### Macrophage polarization

Adherent ESDM and BMDM were primed in vitro to adopt a M1-like or M2-like phenotype by treating with LPS (0.1 µg/mL) (Sigma L4391-1 mg) and IFNγ (10 U/mL) or IL-4 (20 ng/mlL, respectively for 48 h. The Griess Reagent System (Promega) was used to measure NO^−^ levels in culture supernatants according to the manufacturer’s instructions.

### Flow cytometry

Cells were harvested, counted and 1 × 10^6^ cells were resuspended in 100 μL PBS containing 1% Bovine Serum Albumin (BSA). Pre-conjugated antibodies were added at their optimal concentrations and incubated on ice for 20 min. Cells were subsequently washed in 2 mL PBS (containing 1% BSA) and centrifuged at 400×*g* for 5 min. Cell pellets were resuspended in 200 μL PBS with 1% BSA. In most experiments cells were stained with a live/dead cell viability stain such as DAPI or 7-AAD. The following anti-mouse antibodies were used: anti-F4/80 APC (BioLegend), anti-CD11b Alexafluor 488 (BioLegend). The following anti-human antibodies were used: anti-CD93 PE (eBioscience), anti-25F9 APC (eBioscience), anti-CD11b Alexafluor 488 (BioLegend).

### Quantitative real-time PCR (qRT-PCR)

qRT-PCR was performed using the ABI 7500 Fast Real-Time PCR System (Applied Biosystems) and analyzed with SDS software Version 1.4 (Applied Biosystems). The Taqman Fast Universal PCR Master Mix (2×) (Applied Biosystems) was used with UPL assays that were designed on the Universal ProbeLibrarySystem Assay Design Center (Roche) (Supplementary Table 1). The ΔΔCt method was used with GAPDH to normalize cDNA levels and the “control” sample of each experiment was used as a calibrator to calculate relative change in gene expression. All data is presented as fold-change over the expression level of the calibrator.

### Phagocytosis assay by live imaging

1 × 10^5^ macrophages were plated in tissue-culture grade 96-well plates (CellCarrier, PerkinElmer) 3 days prior to imaging. Cells were washed with PBS, 100 μL NucBlue Live ReadyProbes Reagent (Molecular Probes) (two drops per 1 mL PBS) was added to each well and incubated at 37 °C for 20 min. Cells were washed with PBS and treated with 100 μL/well of CellMask^TM^ deep red plasma membrane stain (Molecular Probes) as per the manufacturer’s instructions and incubated for a further 30 min. CellMask^TM^ reagent was aspirated and 100 μL PBS was added to each well. One vial of pHrodo^TM^ Green Zymosan A BioParticles Conjugate (Molecular Probes) was resuspended in 2 mL PBS at 0.5 mg/mL, thoroughly vortexed and briefly sonicated then further diluted 1:5 in PBS. 100 μL of pHrodo^TM^ BioParticles were added to each immediately prior to imaging. Imaging was performed on the Operetta High-Content Imaging System (Perkin Elmer) at 5 min intervals, at 40× magnification, and images were analyzed on the Harmony High-Content Imaging and Analysis Software (Perkin Elmer). Each experiment was carried out three times, and included three technical replicates.

### Liver fibrosis model

Liver injury was induced in 8–10-weeks old 129/SV mice (Harlan Laboratories) by intra-peritoneal injection of 2 μL/g CCl_4_ (1:3 CCL4:olive oil) twice a week over a 4-week period. Mice were randomly selected to receive intravenous injections of ESDMs or control saline (*n* = 5 per group) at the start of week two and mice were culled 21 days later. Livers were perfused with saline, removed and fixed in formalin and processed for immunohistochemistry.

### Liposomal clodronate-mediated Kupffer cell depletion

Liposomal clodronate (5 mg/mL) was purchased from clodronateliposomes.org. The clodronate suspension was brought to 37 °C and mixed thoroughly prior to injecting. 200 μL of liposomal clodronate or control PBS (*n* = 5 per group) was injected into the tail vein.

### Immunohistochemistry

Tissues were embedded in paraffin and 4 μm slices were cut and mounted on glass slides and immunohistochemistry was carried out for detection of αSMA, PanCK, CFSE and F4/80 as described^5, 25^ (See Supplementary Table 2 for antibody details). For PSR staining, sections were dewaxed and rehydrated. Sections were treated in haematoxylin for 8 min followed by PSR for an hour. Slides were subsequently washed in acidified water, dehydrated and mounted.

For quantification of PSR and αSMA staining, images at 40× (Nikon Eclipse E600 microscope) were tiled and quantification of positive staining was carried out using the image analysis software, ImageJ. For quantification of PanCK staining, positive cells were counted from 20 non-overlapping fields per sample as described.^5^

### Statistical analysis

Statistical analysis was performed using the GraphPad Prism software version 6.0c. All data are expressed as mean + standard error of the mean (SEM). *p*-values less than 0.05 were considered statistically significant (*p < 0.05, **p < 0.01, ***p < 0.001, ****p < 0.0001). The following statistical analyses were used: Unpaired Student’s *t*-test and ANOVA.

### Ethical Approval

The animal experiments were approved and conducted in accordance to the UK Home Office regulations (Project Licence No.70/7847).

## Electronic supplementary material

Supplementary Material

Supplementary Figure S5

Supplementary Figure S6

Supplementary Figure S7
