# Author Correction: Injection of embryonic stem cell-derived macrophages ameliorates fibrosis in a murine model of liver injury

**DOI:** 10.1038/s41536-017-0035-y

**Published:** 2017-11-13

**Authors:** Sharmin S. Haideri, Alison C. McKinnon, A. Helen Taylor, Phoebe Kirkwood, Philip J. Starkey Lewis, Eoghan O’Duibhir, Bertrand Vernay, Stuart Forbes, Lesley M. Forrester

**Affiliations:** Centre for Regenerative Medicine, University of Edinburgh, 5 Little France Drive, Edinburgh, EH16 4UU UK

**Correction to:** *npj Regen Med* (2017); doi:10.1038/s41536-017-0017-0; Published online 23 May 2017

The following ethical statement was added to the end of the Methods section in the HTML and PDF versions of this Article: The animal experiments were approved and conducted in accordance to the UK Home Office regulations (Project Licence No.70/7847).

