# Supplementary material for: Injection of embryonic stem cell derived macrophages ameliorates fibrosis in a murine model of liver injury
Source: NPJ Regen Med. 2017 May 23;2:14. doi: 10.1038/s41536-017-0017-0 (PMC5677947; doi:10.1038/s41536-017-0017-0)
Supplement: Supplementary file 1 — Supplementary Material [file 41536_2017_17_MOESM1_ESM.docx]

Supplementary Figure S1

Figure S1: Phagocytosis image analysis using Harmony High Content Imaging and Analysis Software (Perkin Elmer). Photomicrograph showing macrophages stained with Deep Red Plasma Membrane Stain (red plasma membrane), NucBlue Live ReadyProbes reagent (blue nucleus), and pHrodo Green Zymosan Bioparticles (green bioparticles) (A). Blue staining is assigned to nuclei (B), red staining is assigned to cytoplasm (C), and a “cell” is defined as one having a nucleus (blue) and a cytoplasm (red) (D). A cell containing green signal above a certain threshold is recognized as “phagocytic” (green) and a cell without any green signal is recognized as “non-phagocytic” (red) (E). Percentage of phagocytic cells are then plotted for each sample over time (F). See Supplementary Figures S5, 6 and 7 for live videos of naïve, M1 and M2-activated ESDMs respectively.

Supplementary Figure S2


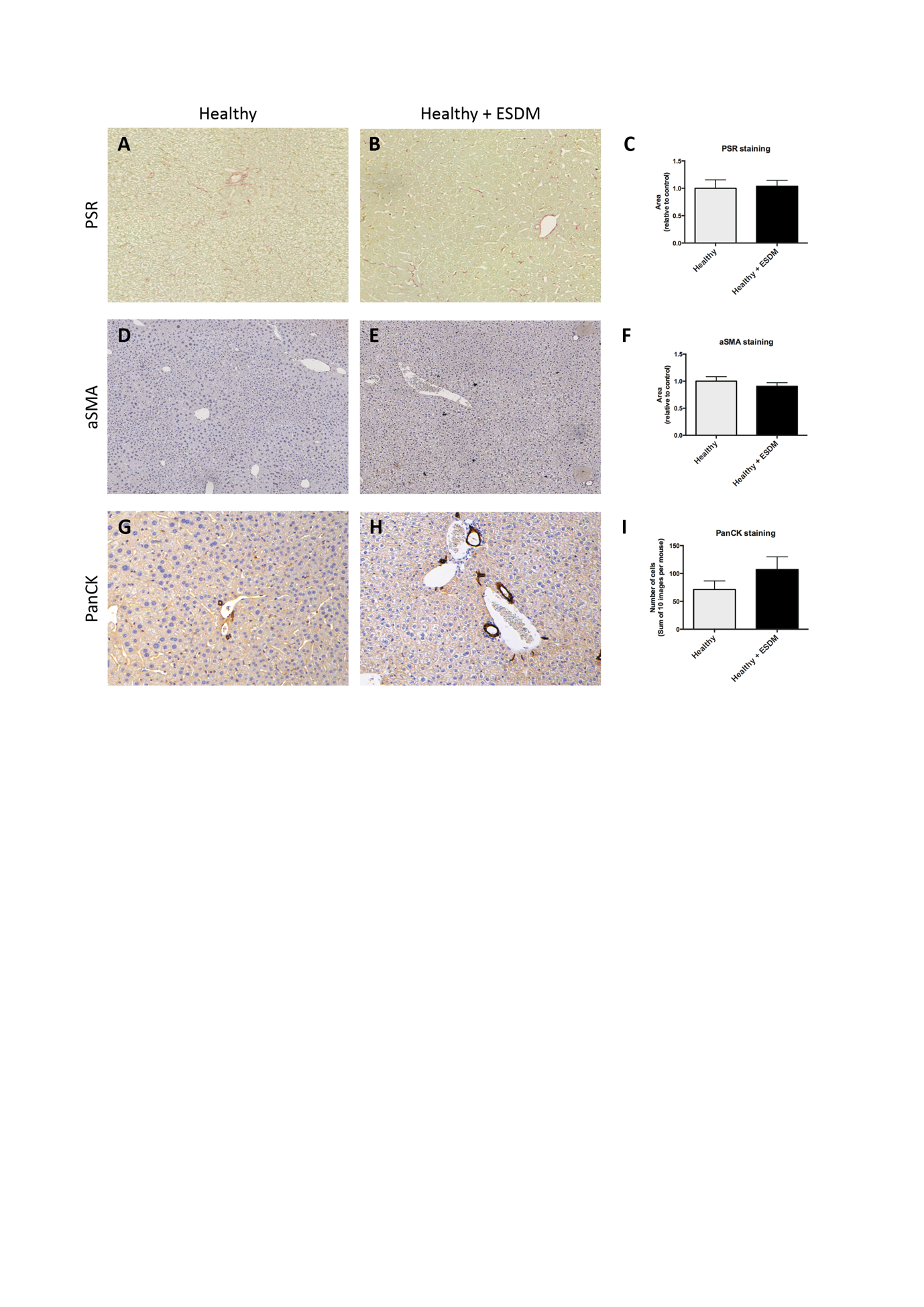


Figure S2: Assessing liver sections for markers of fibrosis upon ESDM delivery. Photomicrograph showing healthy livers stained with PSR, αSMA and PanCK (A, D, G, respectively) and livers 21 days post ESDM delivery (B, E, H, respectively). Quantitative analysis of PSR, αSMA and PanCK positive staining (C,F,I).

Supplementary Figure S3


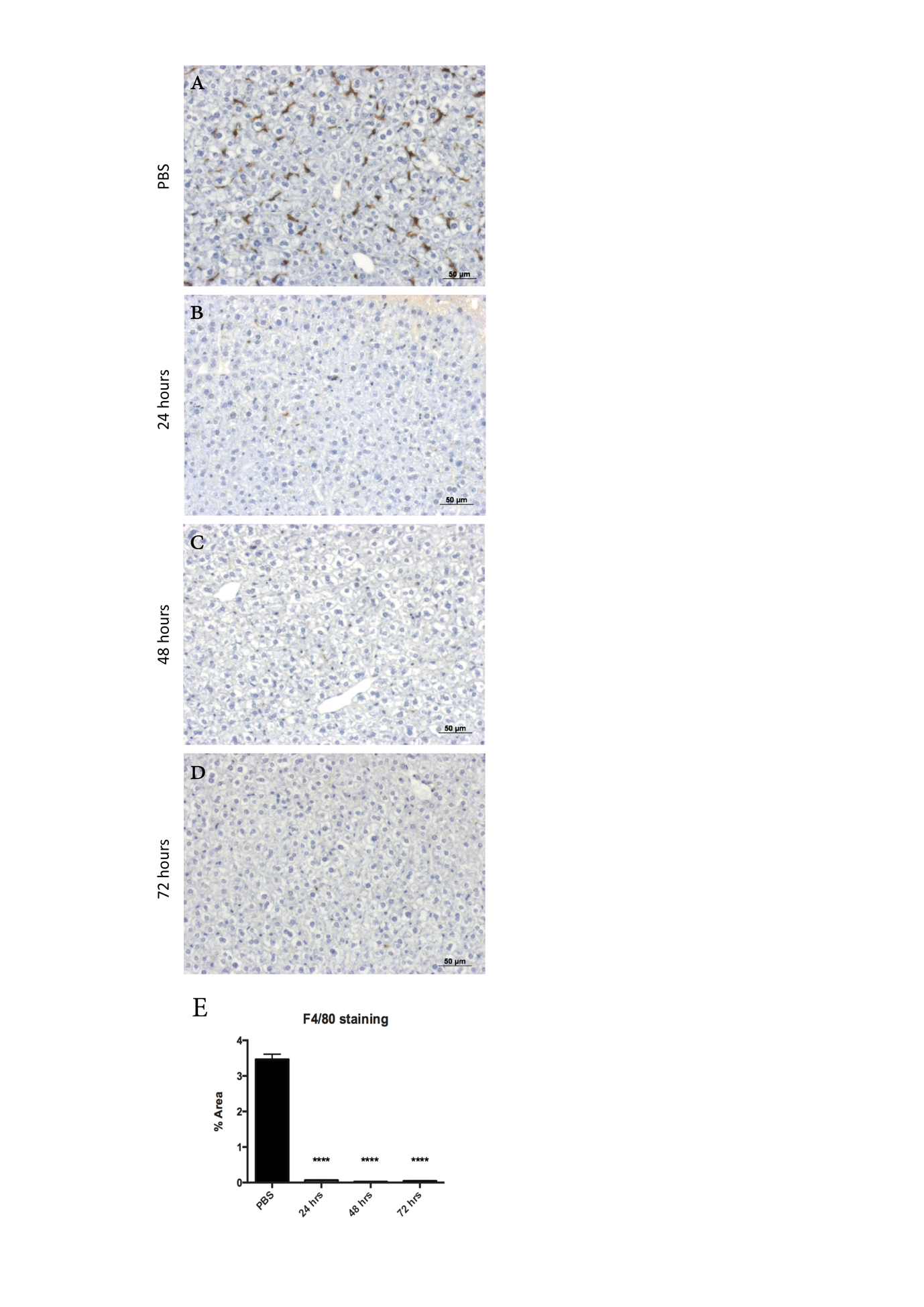


Figure S3: Assessing macrophage populations in livers after liposomal clodronate treatment by F4/80 immunohistochemistry. Representative images of F4/80 stained liver sections of PBS controls (A), 24 hours (B), 48 hours (C), and 72 hours (D) after clodronate treatment. Quantitative analysis of F4/80+ staining (E).

Supplementary Figure S4


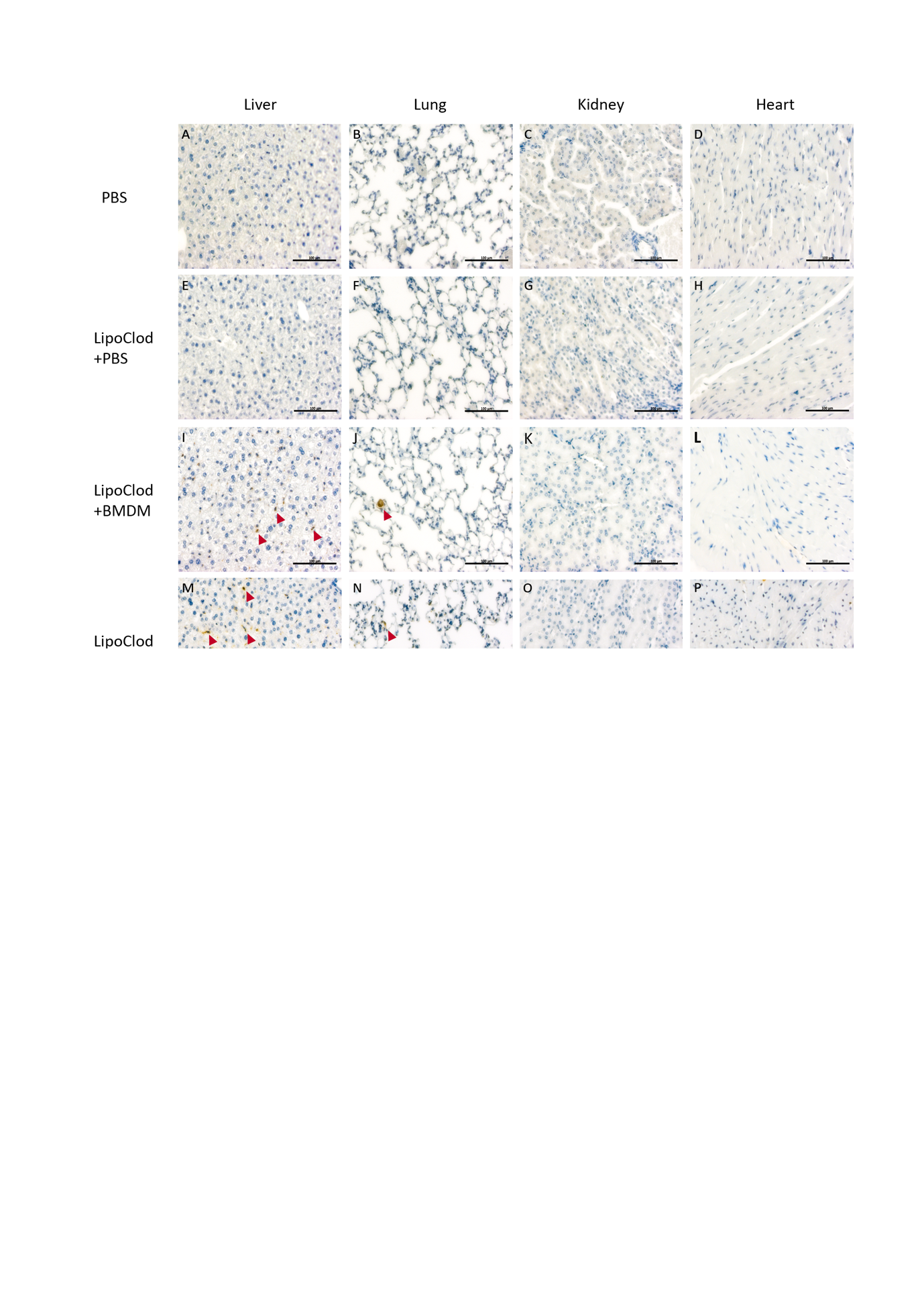
Figure S4: Assessing ESDM and BMDM engraftment in different organs by FITC immunohistochemistry. Representative images of exogenous macrophages in liver, lung, kidney and heart sections of mice treated with PBS (A-D), liposomal clodronate (E-H), liposomal clodronate and BMDM (I-L), and liposomal clodronate and ESDM (M-P), respectively (Scalebars 100 μM).

Supplementary Figure S5

Video of naive murine ESDMs

Supplementary Figure S6

Video of M1-activated murine ESDMs

Supplementary Figure S7

Video of M2-activated murine ESDMs

Supplementary Table 1

**Primer sequences**

| **A** | **Primers for qRT-PCR analysis of mouse macrophage gene expression analysis** | | |
| --- | --- | --- | --- |
|  |  |  |  |
|  | **Gene** | **Forward primer (5'-3')** | **Reverse primer (5'-3')** |
|  | iNOS | AACACCCGGGACATGAGAC | CCTGGGAGGATCAGGAAGTC |
|  | CD86 | GAAGCCGAATCAGCCTAGC | CAGCGTTACTATCCCGCTCT |
|  | ARG1 | GAATCTGCATGGGCAACC | GAATCCTGGTACATCTGGGAAC |
|  | FIZZ1 | TATGAACAGATGGGCCTCCT | AGGCAGTTGCAAGTATCTCCA |
|  | TWEAK | CAGGATGGAGCACAAGCAG | GGCTGGAGCTGTTGATTTTG |
|  | MMP9 | ACGACATAGACGGCATCCA | GCTGTGGTTCAGTTGTGGTG |
|  | MMP12 | GCTGCTCCCATGAATGACA | AAGCATTGCACACGGTTGTA |
|  | MMP13 | GCCAGAACTTCCCAACCAT | TCAGAGCCCAGAATTTTCTCC |
|  |  | | |
|  | **Primers for qRT-PCR analysis of human macrophage gene expression analysis** | | |
| **B** |  |  |  |
|  |  |  |  |
|  | **Gene** | **Forward primer (5'-3')** | **Reverse primer (5'-3')** |
|  | CD40 | GCCAAGAAGCCAACCAATAA | TGGCATCCATGTAAAGTCTCC |
|  | CD80 | AAGCAAGGGGCTGAAAAGAT | AAGGGCAAGGTGGGGTAAT |
|  | TGM2 | TCACCTTCAGTGTCGTGACC | CAGCATCTCTTAGTGGAAAACG |
|  | MRC1 | TGTTTTGGTTGGGATTGACA | TCTCCATAAGCCCAGTTTTCA |

Supplementary Table 2

**List of Antibodies**

| **A** | **Anti-mouse antibodies for flow cytometry** | | | |  |
| --- | --- | --- | --- | --- | --- |
|  |  |  |  |  |  |
|  | **Antibody** | **Fluorochrome** | **Dilution** | **Supplier** |  |
|  | F4/80 | APC | 1/100 | BioLegend |  |
|  | CD11b | Alexafluor488 | 1/100 | BioLegend |  |
|  | SSEA1 | APC | 1/100 | RnDSystems |  |
|  | CSF1R | PE | 1/100 | BioLegend |  |
|  |  |  |  |  |  |
| **B** | **Anti-human antibodies for flow cytometry** | | | |  |
|  |  |  |  |  |  |
|  | **Antibody** | **Fluorochrome** | **Dilution** | **Supplier** |  |
|  | CD43 | PE | 1/100 | eBioscience |  |
|  | CD45 | APC | 5/100 | eBioscience |  |
|  | CD93 | PE | 0.5/100 | eBioscience |  |
|  | CD14 | APC | 5/100 | eBioscience |  |
|  | 25F9 | APC | 4/100 | eBioscience |  |
|  |  |  |  |  |  |
| **C** | **Antibodies for immunohistochemistry** | | | |  |
|  |  |  |  |  |  |
|  | **Antibody** | **Description** | **Dilution** | **Supplier** |  |
|  | aSMA | Mouse anti-mouse | 1/2000 | Ab7817, Abcam | |
|  | PanCK | Rabbit anti-mouse | 1/200 | Z0622, Dako | |
|  | F4/80 | Rat anti-mouse | 1/50 | Ab6640, Abcam | |
|  | FITC | Goat anti-mouse | 1/100 | 71-1900, Invitrogen | |
